# Improving taxonomic classification with feature space balancing

**DOI:** 10.1093/bioadv/vbad092

**Published:** 2023-07-17

**Authors:** Wolfgang Fuhl, Susanne Zabel, Kay Nieselt

**Affiliations:** University of Tübingen, Institute for Biomedical Informatics (IBMI), Sand 14, Tübingen, Baden-Württemberg, 72076, Germany; University of Tübingen, Institute for Biomedical Informatics (IBMI), Sand 14, Tübingen, Baden-Württemberg, 72076, Germany; University of Tübingen, Institute for Biomedical Informatics (IBMI), Sand 14, Tübingen, Baden-Württemberg, 72076, Germany

## Abstract

**Summary:**

Modern high-throughput sequencing technologies, such as metagenomic sequencing, generate millions of sequences that need to be assigned to their taxonomic rank. Modern approaches either apply local alignment to existing databases, such as MMseqs2, or use deep neural networks, as in DeepMicrobes and BERTax. Due to the increasing size of datasets and databases, alignment-based approaches are expensive in terms of runtime. Deep learning-based approaches can require specialized hardware and consume large amounts of energy. In this article, we propose to use *k*-mer profiles of DNA sequences as features for taxonomic classification. Although *k*-mer profiles have been used before, we were able to significantly increase their predictive power significantly by applying a feature space balancing approach to the training data. This greatly improved the generalization quality of the classifiers. We have implemented different pipelines using our proposed feature extraction and dataset balancing in combination with different simple classifiers, such as bagged decision trees or feature subspace KNNs. By comparing the performance of our pipelines with state-of-the-art algorithms, such as BERTax and MMseqs2 on two different datasets, we show that our pipelines outperform these in almost all classification tasks. In particular, sequences from organisms that were not part of the training were classified with high precision.

**Availability and implementation:**

The open-source code and the code to reproduce the results is available in Seafile, at https://tinyurl.com/ysk47fmr.

**Supplementary information:**

Supplementary data are available at *Bioinformatics Advances* online.

## 1 Introduction

Metagenomic analysis of environmental or clinical samples has proven to be highly relevant for characterizing biodiversity (Hoshino *et al.*, 2020; Howe *et al.*, 2014) and has shown that the microbiome has a significant impact on the health of organisms (Rooks and Garrett, 2016; Wade, 2013). The advent of metagenomic sequencing required the development of efficient algorithms to classify the taxonomic origin of sequences and to infer the composition of a microbial community. Taxonomic classification describes the process of assigning a sequence to a specific node in the taxonomy on a pre-defined taxonomic rank, e.g. genus (Menzel *et al.*, 2016).

Taxonomic classification of whole-genome shotgun data is computationally challenging for two main reasons: first, millions of sequences need to be classified, and second, the number of available classes, i.e. reference genomes in a database, is constantly growing. Therefore, fast and resource-efficient algorithms are essential to cope with the amount of data available.

A vast variety of methods have been released, which classify algorithmically into traditional alignment-based and *k*-mer approaches, and more recent methods that apply machine learning (ML) classifiers. BLAST (Altschul *et al.*, 1990) and Diamond (Buchfink *et al.*, 2015, 2021) are famous methods that use seed-and-extend approaches to align the query sequences with a database of microbial genomes. Although precise, these alignment-based tools suffer from a low recall and are relatively slow. To increase speed, methods using local alignments of *k*-mers have been developed, such as Kraken2 (Wood *et al.*, 2019) or MMseqs2 (Steinegger and Söding, 2017). For a more comprehensive review and benchmarking of modern taxonomic classifiers, see Simon *et al.* (2019).

The above approaches rely heavily on a well-curated database and taxonomy. ML classification methods attempt to circumvent this dependency. *k*-mer profiles have been used as features for simple classifiers, such as the Naïve Bayes Classifier (Rosen *et al.*, 2008, 2011; Zhao *et al.*, 2020), or Support Vector Machines (SVMs) (Vervier *et al.*, 2016). However, they have been shown to underperform in terms of precision or to be inefficient in terms of memory or runtime (McIntyre *et al.*, 2017).

Recently, deep neural network (DNN) architectures were developed to capture more complex dependencies between data (sequences) and target variables (taxonomic class). DeepMicrobes (Liang *et al.*, 2020) implements a deep learning architecture consisting of many long short-term memory cells followed by a self-attention mechanism and a multilayer perceptron for classification. DeepMicrobes uses a 12-mer embedding as the first layer of the DNN. BERTax (Mock *et al.*, 2022) uses a transformer model from natural language processing (NLP), trained on large amounts of DNA sequences, to extract a feature representation of the DNA sequence. This feature representation is then fed to a small, fully connected neural network for the final classification. Both approaches, BERTax and DeepMicrobes, show a similar accuracy as alignment-based methods and can be executed extremely fast on GPUs. However, DL methods are often considered black-box approaches that can be difficult to interpret. In addition, the need for specific hardware, e.g. to efficiently train the DNNs, makes these approaches inapplicable for some users.

In this work, we evaluate several pipelines for the taxonomic classification of DNA sequences at three different taxonomic levels (superkingdom, phylum and genus). All pipelines extract *k*-mer profiles from the DNA sequences and use simple ML approaches, such as bagged decision trees or subspace *k*-nearest neighbors (KNNs), for classification. A significant increase in performance could be achieved by applying an algorithm that balances the distribution of training samples across the feature space prior to training.

Compared to alternative approaches, such as BERTax, DeepMicrobes and MMseq2, our pipelines improved classification performance [macro-average precision (MAP)] at phylum level, especially for sequences originating from unseen genera, by ∼15% compared to BERTax. When using closely related species for training, our pipeline significantly outperformed MMseqs2 taxonomy at genus level, improving the performance by ∼11%.

## 2 Methods

### 2.1 Data

Data provided by the developers of BERTax (Mock *et al.*, 2022) were used in this work. The datasets include sequences of length 1500 nt retrieved from archaeal, eukaryotic, viral and bacterial genomes [see Dataset S1 in Mock *et al.* (2022) for accession numbers]. We used a subset of datasets that the authors in Mock *et al.* (2022) referred to as the *distantly related* and the *final model* dataset. Data were downloaded via https://osf.io/qg6mv/ (10.09.22). Training and test set splits were adopted. Table 1 summarizes the characteristics of the datasets. Generally, all classes are covered by at least 10 000 DNA sequences of length 1500 nt.

**Table 1.** Characteristics of the distantly related and the final model dataset provided by Mock *et al.* (2022) that were used in this study

	Distantly related	Final model
No. of classes for		
superkingdom	4	4
phylum	30	43
genus	0	155
Size of		
training set [seqs]	2 245 416	5 311 920
test set [seqs]	53 400	88 000

*Note*: [seqs] refers to the number of sequences of length 1500 nt.

As summarized in Figure 4 in Mock *et al.* (2022), the distantly related and final model datasets were created to stress different aspects of taxonomic classification. In the final model dataset, sequences from the same genus are part of the training as well as the test set. Predicting the genus level is therefore easy because the classifier has seen similar sequences during training. On the other hand, in the distantly related dataset, all sequences originating from one genus are used for either training or testing. When predicting the phylum level of a sequence, the classifier has seen sequences from the same phylum during training, but not from the same genus. This classification task is much more difficult and resembles the classification of novel organisms during metagenomic analysis. In summary, the datasets are designed in a way that test data are more similar to the training data in the final model dataset than in the distantly related dataset due to the selection of samples for each subset.

### 2.2 Feature extraction

From each sample—a DNA sequence of length 1500 nt—a set of features was extracted by calculating the distribution of *k*-mers present in the sequence. To this end, the relative frequency of each of the potentially occurring $4^{k}\;k$-mers is calculated as the quotient of the absolute count and the sum of all *k*-mer counts of the sample. The relative frequency of each *k*-mer is used as a feature.

### 2.3 Dataset balancing

**Figure 1.**
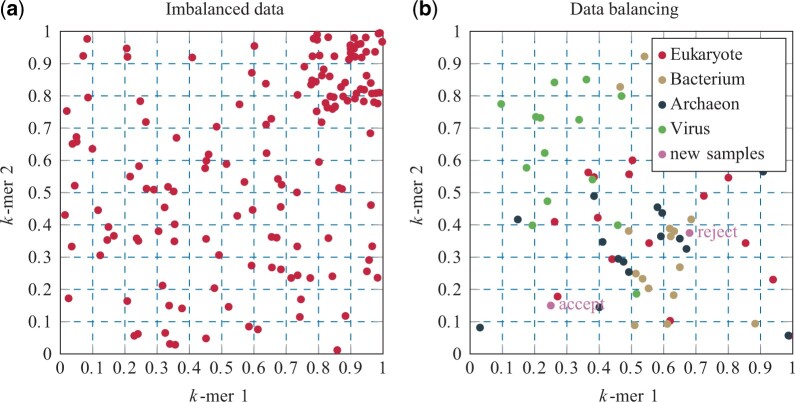
Simplified visualization of the dataset balancing approach. The feature space is considered 2D, where each dimension represents the relative frequency of a specific *k*-mer. Note: For visualization reasons, we neglected that frequencies must add up to one. (**a**) Samples are not uniformly distributed across the feature space. The upper right area contains relatively more samples than the rest of the feature space, thus the dataset is imbalanced. (**b**) Balancing the feature space distribution. The feature space is initialized with 15 samples from all four superkingdom classes. The number of grid cells *G* (per dimension) is set to 10. Due to the current maximal cell count $C_{\text{max}}$ of 5, a new potential next sample is accepted ($1<C_{\text{max}}$) or rejected ($5=C_{\text{max}}$), respectively

After computing the relative *k*-mer distribution from all sequences from the training dataset, the training set is imbalanced since some feature combinations are more prevalent than others. This leads to areas in the feature space that are densely covered, whereas other regions are sparsely occupied (see Fig. 1a). Our approach to dataset balancing aims to establish a more uniform distribution of samples across the feature space. This must not be confused with the commonly known problem of imbalanced classes. To this end, the feature space is discretized using a high-dimensional equidistant grid ($D=4^{k}$). The number of grid cells *G* per feature dimension is a parameter to be learned. The user sets the final number of training samples *N*. After initially adding 0.3⋅N randomly selected samples from all classes to the empty feature space, samples are added to the feature space until *N* samples have been added. Initialization is done to accelerate the balancing algorithm. Adding more samples to the feature space after initialization is shown in Figure 1b and works as follows: let $S_{i}$ be a randomly selected potential new sample from the training data, let *g* be the grid cell of $S_{i}$ in the feature space and let $C_{g}$ be the number of samples already present in the grid cell *g* of $S_{i}$. Further, let $C_{\text{max}}$ be the highest count over all grid cells observed so far. The sample $S_{i}$ is accepted to be added to the grid cell *g* if $C_{g}<C_{\text{max}}$ and rejected if $C_{g}=C_{\text{max}}$. By that, empty areas in the feature space are preferably filled. To prevent the algorithm to get stuck, a sample is accepted independent of $C_{\text{max}}$ after 10 consecutive unsuccessful trials.

### 2.4 ML classifiers

For classification, we used different ML models as ensembles and applied the built-in functions of Matlab 2022b, including bagged decision trees, random subspace *k*-nearest neighbors, random subspace discriminant analysis, SVMs with different kernels (linear, Gaussian and polynomial) and a small neural network with one hidden layer of size 512. In general, hyperparameters were not optimized and default parameters were used.

To evaluate classification performance MAP was used as a metric, which is given by the class-wise average precision where C is the set of all classes, ${\text{TP}}_{i}$ the true-positives of class i∈C and ${\text{FP}}_{i}$ the false-positives of class i∈C.


(1) $$\text{MAP}=\frac{\sum_{i\in C}\frac{{\text{TP}}_{i}}{{\text{TP}}_{i}+{\text{FP}}_{i}}}{\left|C\right|},$$

### 2.5 Used hardware

We used an AMD Ryzen 9 3950 with a 16-Core (32 Threads) processor (3.50 GHz) and 64 GB DDR4 memory to evaluate runtime and resource consumption.

## 3 Results

**Figure 2.**
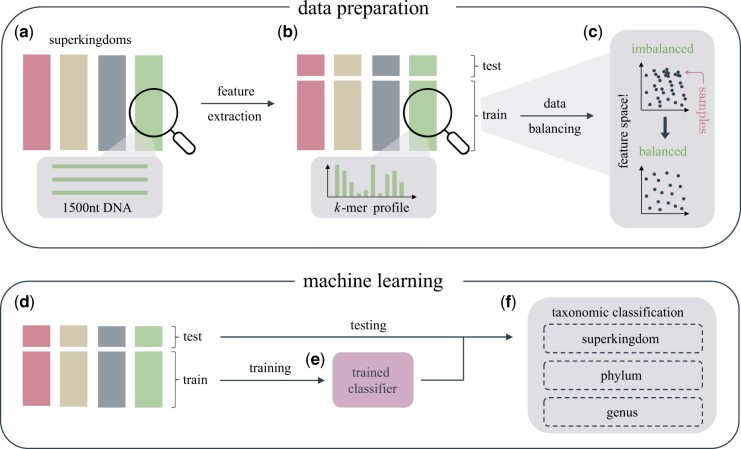
Proposed pipeline. (**a**) Sequences of length 1500 nt originating from four superkingdoms are used as input. (**b**) From each sequence, *k*-mer profiles—the relative frequency of all $4^{k}$ possible words of length *k*—are extracted and used as features. (**c**) Training data are balanced using an undersampling approach. Dense regions of the feature space are thinned out. This reduces the size of the training set. (**d and e**) The balanced and curated training data are used to train simple supervised learning classifiers. (**f**) Depending on the taxonomic rank of the given label, the test sequences are taxonomically classified at the superkingdom, phylum, or genus level

We propose a pipeline for the taxonomic classification of DNA sequences, with the focus of being resource-saving in terms of runtime and memory, while at the same time performing equally well as existing accurate but resource-demanding methods, such as BERTax and DeepMicrobes. To achieve this, simple classification algorithms need to be used instead of complex deep learning methods. However, these simple methods cannot handle unstructured data like e.g. text data. DNA sequences, as typically observed in shotgun sequencing, are highly unstructured because they originate from random locations in the genome, so positional information is not comparable between sequences. We, therefore, extract meaningful features from the DNA sequences to be used as input for the classifiers. Given DNA sequences of length 1500 nt from four superkingdoms—archaea, bacteria, eukaryotes and viruses—(Fig. 2a), we extracted the relative frequency of all *k*-mers, e.g. k=3 results in $4^{3}=64$ possible 3-mers and thus 64 features (Fig. 2b). The choice of the parameter *k* influences the dimensionality *D* of the input data—$D=4^{k}$—and thereby the efficiency of the pipeline.

When inspecting the distribution of the training samples across the $4^{k}$ dimensional feature space, it became apparent that some areas of the feature space are densely covered, whereas other regions are sparsely occupied. As a result, by construction, classifiers focus mainly on the dense regions by fitting complex decision boundaries to the data, which can lead to overfitting in these areas. At the same time, sparse regions become less important, potentially leading to underfitting there. To advance a uniform distribution of samples across the feature space, we applied an undersampling approach keeping most of the data in sparse regions and decreasing the number of samples in dense regions (Fig. 2c). It is important to note that empty regions remain empty. A high-dimensional equidistant grid is used to discretize the feature space since the features (frequencies) are continuous variables. To approximate a uniform distribution, the number of samples in each cell could be counted and samples removed from cells where the cell count exceeds a certain threshold. While being a valid approach, it is unfeasible for large training sets. Therefore, we implemented a time-efficient approximation that allows the user to specify the final training set size *N* in advance. For details see Section 2.4.

The reduced and balanced training set is subsequently used to train simple classifiers and classifier performance is evaluated in terms of MAP on the test dataset (Fig. 2d–f). In various experiments, taxonomic classification is performed at different taxonomic levels, including in order of increasing difficulty, superkingdom, phylum and genus.

In the following sections, the pipeline is applied to and evaluated on two datasets—called distantly related and final model—that were previously published by Mock *et al.* (2022) and contain 1500 nt DNA sequences from four different superkingdoms. For details see Section 2.1.

### 3.1 Dataset balancing improves classification performance

We first evaluated the effect of dataset balancing and different parameter choices of the balancing algorithm—the grid size *G* and the maximal number of training samples *N* kept—on the performance of taxonomic classification. For that, classifiers were trained on the (un)balanced distantly related dataset, and performance is assessed on the test set. All three classifiers (bagged decision tree, subspace KNN and small neural network) increased their classification performance on phylum level as more training samples were added but plateaued at more than $6\times 10^{5}$ training samples (see Supplementary Fig. S1). Therefore, *N* was set to $6\times 10^{5}$ for further experiments.

**Figure 3.**
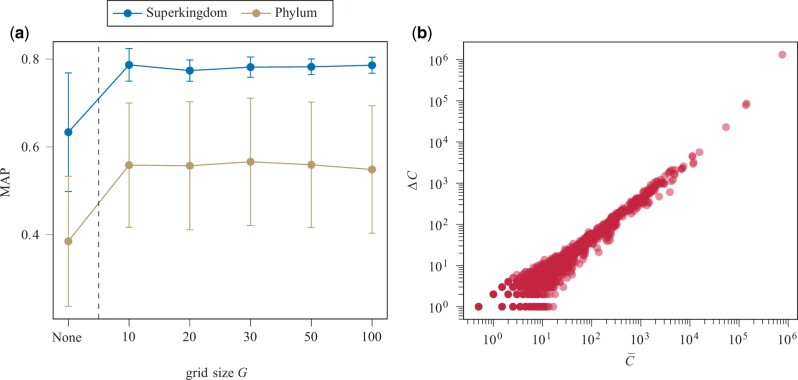
(**a**) Performance evaluation of a classifier (ensemble of bagged decision trees) trained on imbalanced (none) or balanced training data of the distantly related dataset using different grid sizes *G*. Relative *k*-mer frequencies were used as features. Mean MAP values and 1σ-intervals over different choices of k∈{1,2,3,4,5} are shown. (**b**) Effect of data balancing ($N=6\times 10^{5},G=10$) on the sample distribution of the distantly related training set. $\bar{C}=\frac{C_{\text{before}}+C_{\text{after}}}{2}$ describes the average sample count per grid cell before and after data balancing. $\Delta C=C_{\text{after}}-C_{\text{before}}$ describes the number of samples that were removed per grid cell

Figure 3a shows that data balancing improves the classification performance of an ensemble of bagged decision trees. In all experiments, regardless of the choice of *G*, MAP values could be increased by up to 0.18 on average by balancing the training data (see Supplementary Table S1 for exact performance values). Since larger grid sizes increase the complexity of the balancing algorithm, but do not significantly improve the performance, G=10 was chosen for subsequent experiments.

As shown, our approach to data balancing has a positive effect on the performance of taxonomic classification. Reasonable choices have been identified for both *G* and *N*, despite the lack of an exhaustive search for optimal parameters. We used these parameters to balance the distantly related training set. Figure 3b shows a linear increase in the number of training samples removed, ΔC, per grid cell for increasingly dense grid cells, $\bar{C}$, in a log–log plot. This confirms that more training samples are removed in dense regions of the feature space than in sparse regions.

### 3.2 Evaluation of ML methods

To ensure a resource-efficient pipeline, only simple classifiers were considered and their performance was compared. Using default parameters, ensembles of bagged decision trees and ensembles of subspace KNNs classified test data from the distantly related dataset best (Table 2), and were therefore considered for the final evaluations.

**Table 2.** Performance evaluation of different ensemble classifiers trained on the distantly related dataset’s balanced training data ($G=10,N=6\times 10^{5}$)

Classifiers	Superkingdom level	Phylum level
Bagged decision trees	0.8164	**0.6971**
Subspace KNN	**0.8823**	0.6756
Subspace discr. analysis	0.6020	0.4894
Linear SVM	0.6462	0.4505
Gaussian SVM	0.6635	0.5705
Polynomial SVM	0.6737	0.6013
Neural network	0.8121	0.6598

*Note*: Relative 3-mer frequencies were used as features. Classification was performed at the superkingdom and phylum, and performance values are given as MAP. The best results appear in bold.

### 3.3 Evaluation of different *k*-mer sizes

In this section, we show how the size of *k* used to extract relative *k*-mer frequencies from DNA sequences affects classifier performance. To this end, different *k*s and their combinations were evaluated by a 5-fold cross-validation on the training data of the distantly related dataset using ensembles of bagged decision trees and subspace KNNs. As seen in Table 3, the relative frequencies of 3-mers or 4-mers classified sequences best at both taxonomic levels.

**Table 3.** Mean performance of the classifiers subspace KNN and ensemble of bagged decision trees using 5-fold cross-validation on the balanced training data ($G=10,N=6\times 10^{5}$) of the distantly related dataset

	Subspace KNN		Bagged decision trees	
*k*	Superkingdom	Phylum	Superkingdom	Phylum
1	0.5870	0.2588	0.6328	0.3448
2	0.8584	0.6537	0.8664	0.7124
3	0.9438	0.8578	**0.9250**	0.8418
4	**0.9557**	**0.8809**	0.9103	**0.8753**
5	0.9235	0.8326	0.8955	0.8194

*Note*: Relative *k*-mer frequencies were used as features. The first column shows the respective choice of *k*. Classification was performed at the superkingdom and phylum level, and performance values are given as MAP. The best results appear in bold.

### 3.4 Final performance evaluation and comparison to existing methods

**Figure 4.**
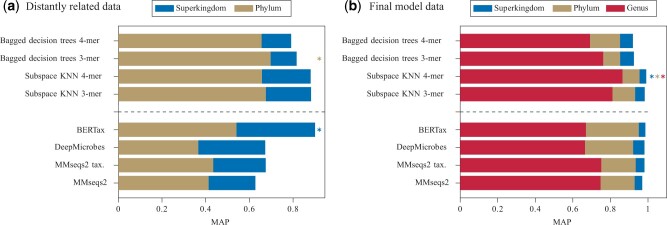
Performance comparison in terms of MAP of several pipelines implementing our approach (upper four bars) with state-of-the-art methods. Note that performance values for state-of-the-art methods were taken from Mock *et al.* (2022). Results for the distantly related (**a**) and final model dataset (**b**) are shown. The best classification performances are labeled by asterisks

In the final evaluation, we compared the accuracy of our pipeline with state-of-the-art approaches for taxonomic classification on both the distantly related and the final model dataset. We chose the methods MMseqs2 (Steinegger and Söding, 2017), DeepMicrobes (Liang *et al.*, 2020) and BERTax (Mock *et al.*, 2022), where MMseqs2 was used in two different modes [MMseqs2 and MMseqs2 taxonomy (Mirdita *et al.*, 2021)]. In the paper by Mock *et al.* (2022), these methods performed best on the dataset that is also used in this article, and are therefore used for comparison with our proposed pipelines. Performance values are shown in terms of MAP in the bottom rows of Figure 4. Exact values are listed in Supplementary Table S2. It should be noted, that the performance values for these methods were not reproduced but copied from Mock *et al.* (2022). However, the same data and dataset splits in training and test sets have been used to make the results comparable. We applied four pipelines that use *k*-mer distributions as features and perform our proposed balancing approach (Fig. 4 and Supplementary Table S2). Overall, all methods performed better on the final model dataset (Fig. 4b) compared to the distantly related dataset (Fig. 4a) on both superkingdom and phylum levels. Test data sequences from the final model dataset are easier to classify because both test and training contain sequences originating from the same genus, whereas in the distantly related dataset sequences from one genus are assigned to either test or training data. It can be further observed that a more specific taxonomic classification is generally more difficult and performance values of all methods drop for phylum (and genus) level classification. When comparing all methods, our proposed ensemble of subspace KNN classifiers on 4-mer distributions outperformed all state-of-the-art methods on the final model dataset (Fig. 4b). Especially, on the genus level a performance increase of 11.34% is denoted compared to the taxonomy mode of MMseqs2. On the distantly related data, all proposed pipelines performed superior to the state-of-the-art methods at phylum level classification with an increase in MAP of up to 15.61%. On the superkingdom level, BERTax outperformed our best result by 1.18% MAP.

We compared the runtime and the memory requirements of MMseq2, BERTax and two of our proposed pipelines to classify the phylum level of all sequences in the distantly related test set on a CPU server with 16 cores, 32 threads and 64 GB of memory (Table 4). Our pipelines took more time than MMseqs2, but less time than BERTax. The ensemble of bagged decision trees was particularly economical in terms of memory consumption. Training our pipelines took 14 and 9 min for subspace KNNs and bagged decision trees, respectively.

**Table 4.** Runtime and memory requirements to classify the phylum level of all test set sequences of the distantly related dataset

Methods	Total memory	Execution
	consumption (GB)	time (s)
MMseqs2	30	55
BERTax	15	3297
Subspace KNN^a^	19	2486
Bagged decision trees^a^	1	335

*Note*: Ensemble subspace KNN and ensemble bagged decision trees were used to classify 3-mer profiles.

a Converted into C++ functions.

## 4 Discussion

ML, and in particular deep learning approaches, has emerged as promising alternatives to alignment-based methods for taxonomic classification. The number of unclassifiable DNA sequences could be reduced by not relying on well-curated databases. Inexplicability and the need for specific hardware (GPUs) to run efficiently are known drawbacks of deep learning approaches. Many tools like Deep Microbes that perform taxonomic classification rely on *k*-mers or their frequencies. More recently, BERTax has been introduced, a DNN using NLP that takes the whole sequence as input. BERTax—as claimed by the authors—has learned the ‘language’ of DNA and outperforms alternative methods in several classification tasks.

This article presents several exemplary pipelines for the taxonomic classification of sequences. Classification at several taxonomic ranks (superkingdom, phylum and genus) is considered. The strength of the pipelines was most evident on a dataset, where samples in the test and training sets were not allowed to be from the same taxonomic genus. All four of our best-performing pipelines significantly outperformed BERTax, DeepMicrobes and MMseqs2 at the phylum level. Therefore, our pipelines seem to work particularly on sequences from novel organisms. In addition, our pipelines were superior to alternative methods for classifying closely related sequences at the genus level, which is considered a more difficult task than classifying higher taxonomic ranks.

Although *k*-mer frequencies have been used by other methods before as features, we could substantially improve their predictive power by combining a training data balancing approach with subspace KNNs and bagged decision trees. We believe that if the training data are more uniformly distributed across the feature space will prevent overfitting in dense areas and underfitting in sparse areas. Undersampling in dense areas significantly increased performance (see Table 3). In addition, reducing the size of the training set makes training faster. Both classifiers, subspace KNNs, and bagged decision trees, use subsampling of features and training samples, respectively, and contribute further to a model that generalizes well. Using *k*-mer frequencies as features has the additional advantage that sequences of any length can be handled within one experiment.

In terms of resources, our proposed pipelines classified sequences faster than BERTax, but slower than MMseq2. Although fast, MMseqs2 required most memory and did not perform well in many classification experiments. Our pipeline that uses bagged decision trees was the most economical in terms of memory. In general, DL models, such as transformer models, are known to be time-consuming to train. However, our pipelines could be trained within minutes (subspace KNNs: ∼14 min, bagged decision trees: ∼9 min).

So far, our pipelines are more of a proof of concept that simple ML can be superior to deep learning approaches for taxonomic classification tasks, and there is room for improvements. Future work could include a more efficient implementation and hyperparameter optimization of the classification methods to further improve performance. In addition, we would like to apply our pipelines to real-world data, e.g. reads obtained from metagenomic analyses, and investigate how our approaches can also be used to increase the taxonomic resolution to species level.

In this article, we show that *k*-mer profiles are predictive features for taxonomic classification, and when used in combination with dataset balancing and simple ML models outperform DL methods. The fact that they are independent of the sequence length and do not require particular hardware are additional features that make the pipelines a promising prototype for further development.

## Supplementary Material

vbad092_Supplementary_Data

## Data Availability

The data underlying this article are available in Seafile, at https://tinyurl.com/ysk47fmr.
