# Supplementary material for: Improving taxonomic classification with feature space balancing
Source: Bioinform Adv. 2023 Jul 17;3(1):vbad092. doi: 10.1093/bioadv/vbad092 (PMC10415173; doi:10.1093/bioadv/vbad092)
Supplement: vbad092_Supplementary_Data [file vbad092_supplementary_data.pdf]

Supplemental Material for the paper “Improving Taxonomic Classification with Feature Space Balancing”

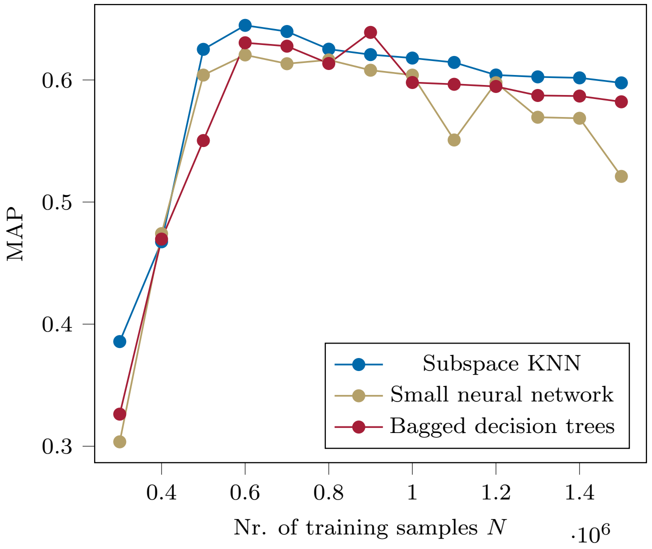

Fig. S1. Performance comparison in terms of MAP for different training set sizes and different classifiers. Performance values for phylum level classification of the distantly related dataset are shown. A grid size G of 10, and k-mer size of 3 was used. The parameter N describes the number of training samples included by the balancing algorithm.

Table S1. Performance evaluation of a classifier (ensemble of bagged decision trees) trained on unbalanced or balanced training data of the distantly related dataset. Relative k-mer frequencies were used as features. The second column shows the respective choice of k. Classification was performed at the superkingdom and phylum level for different grid sizes G, and performance values are given as MAP. The best results appear in bold.

Classification on	k	No balancing	Balancing with				
			$G = 10$	$G = 20$	$G = 30$	$G = 50$	$G = 100$
superkingdom level	1	0.3718	0.7658	0.7661	0.7671	0.7668	0.7667
	2	0.6774	0.7916	0.7909	0.7925	0.7914	0.7902
	3	0.7433	0.8164	**0.8171**	0.8153	0.8149	0.8160
	4	0.7252	0.7911	0.7881	0.7896	0.7912	0.7877
	5	0.6496	0.7693	0.7069	0.7439	0.7478	0.7693
phylum level	1	0.1267	0.2834	0.2840	0.2843	0.2849	0.2835
	2	0.4362	0.5829	0.5783	0.5816	0.5835	0.5828
	3	0.5397	**0.6971**	0.6910	0.6928	0.6964	0.6936
	4	0.4982	0.6562	0.6282	0.6582	0.6605	0.6452
	5	0.3242	0.5728	0.6036	0.6134	0.5708	0.5372

Table S2. Performance comparison in terms of MAP of several pipelines implementing our approach to state-of-the-art methods. Note that performance values for state-of-the-art methods were taken from [Mock et al., 2022]. Different combinations of the proposed approach are shown. The best results appear in bold.

		Distantly related		Final model		
	Methods	Superkingdom	Phylum	Superkingdom	Phylum	Genus
State-of-the-art (numbers taken from Mock et al. [2022])	MMseqs2	0.6276	0.4136	0.9694	0.9290	0.7476
	MMseqs2 tax.	0.6747	0.4344	0.9811	0.9347	0.7509
	DeepMicrobes	0.6725	0.3661	0.9813	0.9211	0.6643
	BERTax	**0.9006**	0.5410	0.9862	0.9510	0.6692
Proposed	Subspace KNN, 3-mer	0.8823	0.6756	0.9828	0.9316	0.8108
	Subspace KNN, 4-mer	0.8803	0.6577	**0.9907**	**0.9553**	**0.8643**
	Bagged decision trees, 3-mer	0.8164	**0.6971**	0.9251	0.8517	0.7610
	Bagged decision trees, 4-mer	0.7911	0.6562	0.9198	0.8513	0.6911
	Chance level	0.25	0.0333	0.25	0.0227	0.0064

References

F. Mock, F. Kretschmer, A. Kriese, S. Böcker, and M. Marz. Taxonomic classification of dna sequences beyond sequence similarity using deep neural networks. *Proceedings of the National Academy of Sciences*, 119(35):e2122636119, 2022.
